# AGE-FRIENDLY UNIVERSITIES: POSSIBILITIES AND POWER IN CAMPUS CONNECTIONS

**DOI:** 10.1093/geroni/igz038.1576

**Published:** 2019-11-08

**Authors:** Joann M Montepare

**Affiliations:** Lasell College, Newton, Massachusetts, United States

## Abstract

Populations are aging locally, nationally, and globally – and challenging institutions of higher education to consider how they can respond to these changing demographics through new approaches to teaching, research, and community engagement. The Age-Friendly University (AFU) initiative was recently launched by an international team convened by Dublin City University, and endorsed by the Academy for Gerontology in Higher Education (AGHE). The AFU concept and 10 guiding principles provide a guiding campus-wide framework that colleges and universities can use for distinguishing and evaluating age-friendly programs and policies, as well as identifying institutional gaps and opportunities for growth. To date, over 45 institutions have joined the AFU global network. This presentation will describe how collaborations across aging-focused programs and campus units devoted to diversity, community engagement, professional studies, and related educational efforts offer prime opportunities to build and sustain an AFU vision.

